# CLU, CR1 and PICALM genes associate with Alzheimer's-related senile plaques

**DOI:** 10.1186/alzrt71

**Published:** 2011-04-05

**Authors:** Eloise H Kok, Teemu Luoto, Satu Haikonen, Sirkka Goebeler, Hannu Haapasalo, Pekka J Karhunen

**Affiliations:** 1 School of Medicine, University of Tampere, Medisiinarinkatu 3, 33014 Tampere, Finland; 2 National Institute for Health and Welfare, Biokatu 10, 33520 Tampere, Finland; 3 Central Laboratory, Tampere University Hospital, Biokatu 4, 33521 Tampere, Finland

## Abstract

**Introduction:**

*APOE *is the strongest risk gene for sporadic Alzheimer's disease (AD) so far. Recent genome wide association studies found links for sporadic AD with *CLU *and *CR1 *involved in Aβ clearance, and *PICALM *affecting intracellular trafficking.

**Methods:**

We investigated the associations of senile plaques (SP) and neurofibrillary tangles (NFT) with the proposed risk genes and *APOE*, in the Tampere Autopsy Study (TASTY) series (603 cases), a sample of the general population (0 to 97 yrs), who died out-of-hospital.

**Results:**

Age and the *APOE*ε4 allele associated strongly with all phenotypes of SP, as expected. In age and *APOE*ε4 adjusted analyses, compared to the most common homozygous genotype, burnt out SP were more common among carriers of the C-allele of *CLU*, whereas the T-allele of *PICALM *and C-allele of *CR1 *were linked with lower SP coverage. We found no significant associations between any of the genetic variants and NFT.

**Conclusions:**

Marginal effects from *CLU*, *CR1 *and *PICALM *suggest that these genes have minimal effects on the development of AD lesions.

## Introduction

Alzheimer's disease (AD) is the most common form of dementia in Western society and is, and will continue to be, a burden on health systems in the future as the population ages. Age is the largest risk factor for the disease, with higher incidences in older populations [1,2].

Identification of genes related to sporadic AD risk has been slow with study groups isolating only one strongly associated gene: *APOE *[3,4]. The epsilon 4 allele of apolipoprotein E (*APOE*ε4) provides odds ratios (ORs) of between 3 and 25 [5,6] for disease association. APOEε4 is suspected to have a lower effectiveness at transporting cholesterol and is not as efficient at repairing neuronal damage as APOEε3 [7]. One or even two copies of the allele, however, are not sufficient to cause the disease, as many carriers of two ε4 alleles do not develop AD [5].

Studies aiming to detect genes associated with disease risk have used heterogeneous AD cohorts and ascertained few polymorphisms with only a minor impact on disease incidence. One of the problems is to distinguish between pure AD, vascular dementia and other dementia types in clinical cohorts [8-10]. The only consistent and currently accepted method for confirming AD is with post-mortem assessment of the neuropathological lesions neurofibrillary tangles (NFT) and senile plaques (SP) [11-14].

Demented individuals do not always exhibit large enough numbers of SP to warrant an AD diagnosis [15] and NFT and SP are both relatively common in the general population [16-19]. Furthermore, these lesions do not provide a clear-cut explanation as to the cause of AD, with different theories advocating amyloid beta (Aβ) accumulation [12,20] or hyperphosphorylated NFT-causing tau protein [21] as the underlying initiating mechanisms that trigger the disease.

Two recent extensive genome-wide association studies (GWAS), comprising 12,000 probable AD cases and 18,000 age-matched non-demented controls [22,23], revealed three new candidates for genetic risk of developing late onset or sporadic AD: *CLU*, *CR1 *and *PICALM*. Phosphatidylinositol-binding clathrin assembly protein (PICALM) is involved in synaptic neurotransmitter release and intracellular trafficking [24-26], whilst complement component (3b/4b) receptor 1 (CR1), the main receptor of complement C3b protein, binds Aβ and thus may promote clearance [27-30]. Clusterin (CLU, and also known as ApoJ), was replicated independently in the two studies and is thought to bind and remove Aβ from the brain, as well as assist in re-entry of Aβ [31-34].

We have previously shown that as many as one-third of non-demented individuals in an autopsy series-based sample carry SP and more than 40% NFT, with strong age dependence [16]. This suggests that in clinical study series, non-demented control patients may not be free of AD-related neuropathological lesions. Utilising this same cohort, we aimed to investigate whether SP and NFT are associated with any of the recently identified GWAS single nucleotide polymorphisms (SNPs); *CLU*, *CR1 *and *PICALM *to examine their involvement in the development of these brain lesions.

## Materials and methods

### Autopsy series

The Tampere Autopsy Study (TASTY) cohort consisted of 603 autopsy cases, of which the majority died out-of-hospital within Tampere, Finland and surroundings, collected during the years 2002 to 2004 (described in detail elsewhere [16]). The study was approved by the Board of Medicolegal Affairs of Finland. Females within the cohort accounted for 35.8% and the ages for the entire series ranged from 0 to 97, with an average of 63 years (59 years for males and 68 years for females). Of the cases, 6 (1%) had a clinical AD diagnosis, 16 (2.7%) undefined dementia, 10 (1.7%) had memory disorders and 1 (0.2%) had Parkinson's disease prior to death (according to available hospital records and next of kin reports). In some cases it was impossible to obtain all variables due to technical difficulties and sample damage.

### Alzheimer-related lesion measurements

SP and NFT staining and measures have be portrayed previously [16]. Briefly, the Bielschowsky argyrophilic silver impregnation method was performed on samples and measured by two researchers to acquire SP (neocortex) and NFT (hippocampus) counts. Each area was screened to find the highest density of SP (neocortical area at 100 × magnification) and NFT (hippocampus - CA1 area at 200 × magnification) and then scored using a square microscopic grid (SP - 100 intersections covering 1 mm^2^, NFT - four to six random columns), before creating an average percentage of coverage (SP) or average number in 1 mm^2 ^(NFT). Bielschowsky staining was highly correlated with Amyloid beta (Aβ) staining (antibody from Cell Signaling Technology, Danvers, MA, USA; concentration 1:150), which was used to verify our previous results [16], and used the same cortical coverage of SP method. SP and NFT variables included the following categorisations as measured by a neuropathologist: SP (No, Yes), SP type (No Plaques, Diffuse, Primitive, Classic, Burnt Out), SP type 2 (No Plaques, Non-neuritic SP, Neuritic SP), NFT (No, Yes), where reference groups were those with 'No SP' or 'No NFT' and those with either brain lesion were considered 'affected'. Semi-quantitative data for SP utilised the categories 'no', 'sparse', 'moderate' and 'frequent' SP.

### Genotyping

The ABI Prism 7900HT Sequence Detection System used 1 μl DNA with PCR primers (Applied Biosystems, Espoo, Finland) for rs11136000 (*CLU*), rs1408077 (*CR1*) and rs3851179 (*PICALM*). All SNPs were in Hardy-Weinberg equilibrium and genotyping confirmed using SDS version 2.2 (Applied Biosystems). Genotyping for *APOE *has been previously described [16]. Genotyping for the polymorphisms of *CLU*, *CR1 *and *PICALM *were successful for 94%, 97% and 97% of the TASTY cases, respectively.

### Statistics

Logistic regression analyses, with continuous age and *APOE*ε4 carriership as covariates (where possible), were used with SPSS (version 14.0 for Windows; SPSS Finland Oy, Espoo, Finland) to determine associations between the SNPs and AD-related neuropathological lesions. For all SNPs, the most common homozygous genotype was used as the reference group. As previously mentioned, those unaffected by SP or NFT were considered the reference group for the brain lesion categories. When analysing with the cohort split by age groups, the following categories were used: 0 to 49 years, 50 to 59 years, 60 to 69 years, 70 to 79 years, 80+ years, with the youngest group (0 to 49 years) considered the reference group with respect to age, in analyses. The cohort was also split by gender, where mentioned.

## Results

### Autopsy series characteristics

The Tampere Autopsy Series (TASTY) (*n *= 603) comprises consecutive autopsies on males and females aged 0 to 97 years that lived outside institutions or hospitals (see Table 1). Females were on average 10 years older than males, but males were more likely to have SP compared to females (odds ratio (OR) 2.15, *P *< 0.0001, 95% confidence intervals (CI) 1.49 to 3.11). When age was divided into five equal-sized groups, each age group was consistently more likely to have SP compared to the youngest group, with each association also highly statistically significant (see Table 2). This was also true for NFT prevalence (see Table 2), with females more likely than males to have NFT (OR 2.18, *P *< 0.0001, CI 1.49 to 3.18).

**Table 1:** TASTY cohort characteristics

Age (years)	0 to 97	62.67
Males	388	64.3%
Cause of Death		
Disease	59.1%	
Accident	26.8%	
Suicide	11.8%	
Homicide	0.5%	
Unknown	1.5%	
Dementia Status		
AD	6	1%
Dementia	16	2.7%
Memory Problems	10	1.7%
Parkinson's Disease	1	0.2%
SP	172	31.1%
NFT	204	42.1%
SP type		
Diffuse	21	3.7%
Primitive	35	6.2%
Classic	83	14.7%
Burnt Out	25	4.4%
SP type 2		
Non-neuritic SP	56	9.9%
Neuritic SP	108	19.1%
Semi quantitative SP coverage		
Sparse SP	90	16.2%
Moderate SP	62	11.2%
Frequent SP	32	5.8%
*APOE*ε4 carriership	187	31.1%

**Table 2:** Senile plaque and neurofibrillary tangle prevalence in the TASTY cohort by age group

Age group (years)	Total	Affected (%)		*P-*value	Odds Ratio	95% Confidence Interval
Senile plaques						
0 to 49	119	7	5.9	Ref	-	-
50 to 59	101	17	16.8	0.013	3.24	1.29 to 8.16
60 to 69	89	21	23.6	0.001	4.94	1.99 to 12.24
70 to 79	130	56	43.1	<0.0001	12.11	5.23 to 28.01
80+	114	71	62.3	<0.0001	26.42	11.27 to 61.96
Neurofibrillary tangles						
0 to 49	103	13	12.6	Ref	-	-
50 to 59	90	28	31.1	0.002	3.13	1.50 to 6.51
60 to 69	82	23	28.0	0.010	2.70	1.27 to 5.74
70 to 79	109	62	56.9	<0.0001	9.13	4.56 to 18.28
80+	100	78	78.0	<0.0001	24.55	11.60 to 51.95

Those 'Affected' refers to those cases in the TASTY cohort that are affected by senile plaques or neurofibrillary tangles, accordingly.

### *APOE*, *CLU*, *CR1* and *PICALM* associations with SP

As expected, *APOE*ε4 carriership was significantly associated with increased risk of having SP (OR 2.52, *P *< 0.0001, CI 1.72 to 3.68); having both non-neuritic (OR 2.42, *P *= 0.003, CI 1.36 to 4.29) and neuritic SP (OR 2.58, *P *< 0.0001, CI 1.66 to 4.02) compared to no SP; and having primitive (OR 2.53, *P *= 0.010, CI 1.25 to 5.10), classic (OR 2.52, *P *< 0.0001, CI 1.54 to 4.13) and burnt out SP (OR 2.77, *P *= 0.014, CI 1.22 to 6.27) compared to no SP, when evaluated against non ε4 carriers (see Table 3). Results showed similar trends when the cohort was split by gender (data not shown).

**Table 3:** Association of senile plaque type with *APOE*, *CLU*, *CR1 *and *PICALM *genotypes

		SP			Diffuse			Primitive			Classic			Burnt out		
						
	Valid N	SP	OR (95% CI)	*P-*value	Diffuse	OR (95% CI)	*P-*value	Primitive	OR (95% CI)	*P-*value	Classic	OR (95% CI)	*P-*value	Burnt out	OR (95% CI)	*P-v*alue
*APOE*ε4-^1^	388	88 (22.7)	-	-	12 (3.1)	-	-	19 (4.9)	-	-	44 (11.3)	-	-	13 (3.4)	-	-
*APOE*ε4+	174	74 (42.5)	3.2 (2.05 to 4.90)	<0.0001*	9 (5.2)	2.4 (0.97 to 5.86)	0.059	16 (9.2)	2.8 (1.37 to 5.74)	0.005*	37 (21.3)	3.2 (1.83 to 5.48)	<0.0001*	12 (6.9)	3.8 (1.56 to 9.01)	0.003*
																
*CLU *TT^2^	336	89 (26.5)	-	-	13 (3.9)	-	-	22 (6.5)	-	-	48 (14.3)	-	-	6 (1.8)	-	-
*CLU *C+	194	60 (30.9)	1.1 (0.73 to 1.76)	0.570	6 (3.1)	0.79 (0.29 to 2.14)	0.641	11 (5.7)	0.9 (0.39 to 1.84)	0.680	27 (13.9)	0.99 (0.56 to 1.77)	0.988	16 (8.2)	4.4 (1.61 to 12.2)	0.004*
																
*CR1 *AA^2^	186	56 (30.1)	-	-	8 (4.3)	-	-	9 (4.8)	-	-	28 (15.1)	-	-	11 (5.9)	-	-
*CR1 *C+	361	102 (28.3)	0.9 (0.62 to 1.54)	0.924	12 (3.3)	0.71 (0.28 to 1.80)	0.475	24 (6.6)	1.2 (0.55 to 2.77)	0.611	53 (14.7)	0.85 (0.48 to 1.49)	0.572	13 (3.6)	0.57 (0.24 to 1.40)	0.221
																
*PICALM *CC^2^	219	71 (32.4)	-	-	7 (3.2)	-	-	19 (8.7)	-	-	37 (16.9)	-	-	8 (3.7)	-	-
*PICALM *T+	327	87 (26.6)	0.6 (0.41 to 0.95)	0.028*	12 (3.7)	1.09 (0.42 to 2.84)	0.864	16 (4.9)	0.5 (0.26 to 1.09)	0.086	42 (12.8)	0.73 (0.42 to 1.25)	0.253	17 (5.2)	1.36 (0.55 to 3.39)	0.508

Abbreviations: *APOE *, Apolipoprotein E; CI, confidence intervals; *CLU *, Clusterin; *CR1*, complement component (3b/4b) receptor 1; N, number of cases; OR, odds ratio; *PICALM*, phosphatidylinositol-binding clathrin assembly protein; SP, senile plaques; No SP was the reference group. Numbers in brackets are percentages, unless otherwise stated. In some cases it was impossible to obtain all variables due to technical difficulties and sample damage.

* Denotes statistically significant values.

^1 ^Denotes reference group used for statistical analyses. Age was used as a covariate.

^2 ^Denotes most common homozygous genotype and used as the reference group for statistical analyses. *APOE*ε4 carriership and age were used as covariates.

**Figure 1:**
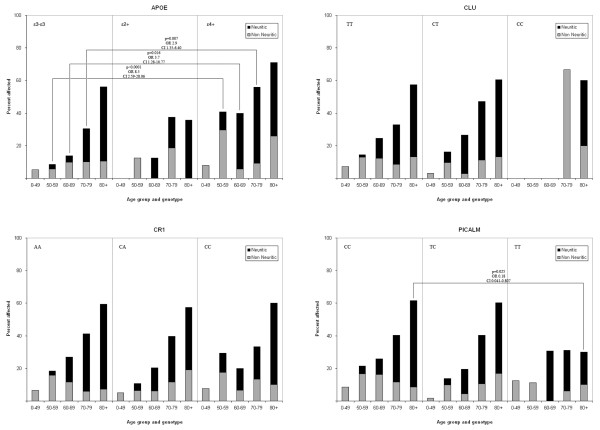
**Senile plaque prevalence by age and genotype (*APOE, CLU, CR1 *and *PICALM*)**. CI = confidence interval; OR = odds ratio.

*APOE*ε4 carriers, compared to ε3-ε3 carriers, were significantly associated with an increased risk of having SP in all age groups except the youngest and oldest (Figure 1). There was a trend of age-related increases in SP, especially of the neuritic type, across all studied genotypes. The *APOE*ε2 carrier group was too small to investigate supposed protective effects, although previously published results suggest tendencies towards protection [16]. In *APOE*ε4 adjusted analyses, 80+ year old carriers of the rare TT genotype of *PICALM *had a significantly lower incidence of SP compared to the common CC carriers (OR 0.18, *P *= 0.025, CI 0.04 to 0.81) (see Figure 1). This association was not seen among younger age groups. There were no significant associations between genotypes of *CLU *and *CR1 *and SP prevalence.

Grouping the rare homozygote and heterozygotes versus the common homozygotes for the SNPs uncovered statistically significant associations between the T allele of *PICALM *and SP (OR 0.62, *P *= 0.028, CI 0.41 to 0.95, versus CC genotype). When we divided the SP into diffuse, primitive, classic and burnt out phenotypes (to investigate the particular phases of the SP life cycle), we found that the rare C allele of *CLU *was significantly associated with the presence of late stage SP (OR 4.4, *P *= 0.004, CI 1.61 to 12.2) compared to the common TT genotype (Table 3). In that setting, the statistically significant association of the *PICALM *T allele was lost.

### *APOE*, *CLU*, *CR1* and *PICALM* associations with SP frequency

When analyses were performed with SP frequency as the dependent variable, *APOE*ε4 carriership was again found to be highly significantly associated with increasing SP coverage, compared to ε3-ε3 carriers (see Table 4). *PICALM *TC genotypes (versus CC genotype) were significantly less likely to have moderate SP compared to no SP (OR 0.42, *P *= 0.012, CI 0.21 to 0.83), whilst *CR1 *CC genotype carriers (compared to AA genotype) were more likely to have sparse SP than no SP (OR 2.1, *P *= 0.048, CI 1.01 to 4.43).

**Table 4:** Association of senile plaque coverage with *APOE*, *CLU*, *CR1 *and *PICALM *genotypes

			Sparse SP			Moderate SP			Frequent SP		
					
	Valid N	No SP	Sparse SP	OR (95% CI)	*P-*value	Moderate SP	OR (95% CI)	*P-*value	Frequent SP	OR (95% CI)	*P-v*alue
*APOE*ε4-^1^	378	281 (74.3)	51 (13.5)	-	-	29 (7.7)	-	-	17 (4.5)	-	-
*APOE*ε4+	174	89 (51.1)	39 (22.4)	2.86 (1.73 to 4.74)	<0.0001*	32 (18.4)	5.32 (2.81 to 10.06)	<0.0001*	14 (8.0)	3.97 (1.76 to 8.94)	0.001*
											
*CLU *TT^2^	329	227 (69.0)	48 (14.6)	-	-	33 (10.0)	-	-	21 (6.4)	-	-
*CLU *C+	192	123 (64.1)	37 (19.3)	1.26 (0.76 to 2.09)	0.378	23 (12.0)	1.06 (0.55 to 2.03)	0.873	9 (4.7)	0.68 (0.29 to 1.63)	0.391
											
*CR1 *AA^2^	182	125 (68.7)	22 (12.1)	-	-	21 (11.5)	-	-	14 (7.7)	-	-
*CR1 *C+	355	234 (65.9)	65 (18.3)	1.48 (0.85 to 2.58)	0.167	38 (10.7)	0.92 (0.48 to 1.78)	0.807	18 (5.1)	0.62 (0.28 to 1.38)	0.242
											
*PICALM *CC^2^	217	136 (62.7)	33 (15.2)	-	-	32 (14.7)	-	-	16 (7.4)	-	-
*PICALM *T+	319	222 (69.6)	54 (16.9)	0.97 (0.58 to 1.60)	0.898	27 (8.5)	0.43 (0.23 to 0.82)	0.010*	16 (5.0)	0.58 (0.26 to 1.26)	0.169

Abbreviations: *APOE *, Apolipoprotein E; CI, confidence intervals; *CLU *, Clusterin; *CR1*, complement component (3b/4b) receptor 1; N, number of cases; OR, odds ratio; *PICALM*, phosphatidylinositol-binding clathrin assembly protein; SP, senile plaques. No SP was the reference group. Numbers in brackets are percentages, unless otherwise stated. In some cases it was impossible to obtain all variables due to technical difficulties and sample damage.

* Denotes statistically significant values.

^1 ^Denotes reference group used for statistical analyses. Age was used as a covariate.

^2 ^Denotes most common homozygous genotype and used as the reference group for statistical analyses. *APOE*ε4 carriership and age were used as covariates.

When we grouped the rare homozygote and heterozygotes together versus the common homozygotes (Table 4), significance was lost for *CR1*, however *PICALM *T allele carriers remained less likely to have coverage of SP versus no SP compared to CC genotype, however again statistical significance was only reached for moderate SP (OR 0.43, *P *= 0.010, CI 0.23 to 0.82).

### Associations with gender

Reanalysing the significant associations with the cohort split by gender gave similar results (data not shown), with females generally more strongly associated, most likely due to their older age. No further significant associations were uncovered.

### Associations with Aβ staining

**Figure 2:**
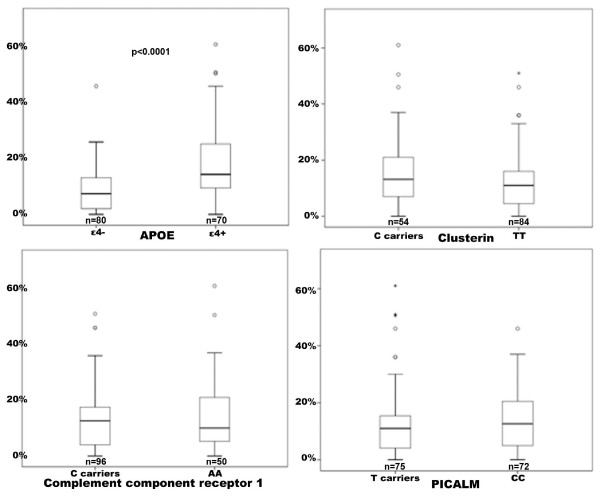
**Boxplots of cortical SP coverage (%) according to Aβ staining and genotype (*APOE, CLU, CR1 *and *PICALM*)**.

A subpopulation of the cohort were assessed for associations with immunohistochemical staining (*n *= 152). None of the newly identified SNPs were statistically significantly associated with Aβ staining, as seen in Figure 2. *APOE*ε4 carriership, however, was significantly associated with higher cortical coverage of Aβ staining (*P *< 0.0001).

## Discussion

AD is the most common form of dementia, but to date its aetiology has remained elusive, despite intensive research. The proposed causes of AD relate to neuropathological findings post-mortem, which is the only way to definitively confirm a patient's diagnosis [11-14]. Diagnosis of the first AD patient, back in 1906, revealed large numbers of SP and NFT; however, although new treatments aimed at reversing the disease by reducing SP have proven successful, they have been without improvements in cognitive abilities of patients [35]. Furthermore, studies have shown cognitively normal elderly can also have large numbers of these brain lesions [16-19] and not all AD cases have the required amounts to corroborate cognitive dysfunction [15].

Genome wide association studies (GWAS) investigating AD have in the past not been powerful enough to reveal anything except *APOE*. Two recent large GWAS [22,23], however, collectively investigated over 30,000 individuals (with almost 12,000 probable AD cases) and examined around 500,000 SNPs that may influence AD risk.

We recently showed that SP and NFT were surprisingly common in a non-demented autopsy series, which represents the closest model to a population sample and that the occurrence of SP, but not NFT, was strongly affected by the *APOE*ε4 allele, regardless of age [16]. Because of the GWAS' discoveries of three potential new candidates for AD risk, we decided to look at their associations with the neuropathological lesions SP and NFT in our cohort to investigate their involvement in the development of these brain lesions.

SP associated with both age and gender, and the *APOE*ε4 allele was highly associated with SP in many of our analyses. Additional analyses showed that the *APOE*ε4 associations were extremely robust in the TASTY series, thus validating our cohort's ability to detect associations with the measured brain lesions. However, whilst NFT were found to associate with age and gender, they were not associated with any of the SNPs investigated. The strong association seen between males and SP prevalence, and females and NFT occurrence may be a confounding factor, due to the older average age of females in the cohort. Additionally, our cohort may over-represent early and violent deaths; however, all cases were included to best represent a population-based investigation.

We hypothesised that the three other SNPs (*CLU*, *CR1 *and *PICALM*) would also associate with SP, as they are involved in AD pathways and most likely would be associated with the development of brain lesions [24-34]. Our results indicate that we did not find as many robust correlations as for *APOE*.

Genetic variants of *CLU*, *PICALM *and *CR1 *genes were associated with SP and remained so with the inclusion of *APOE*ε4 carriers and age as covariates. The appearance of an increased risk for *CLU *C carriers versus TT is unusual in that it only applies for Burnt Out SP - a group in the cohort that is relatively small and are majority females. This suggests that the effect of *CLU *could be on the later stages of SP development and related to removal of Aβ [31-34] being reduced in efficiency.

The *PICALM *T allele appears to have a protective effect on SP prevalence, true also for TT genotypes, versus CC genotypes in the oldest age group. This may be due to more efficient intracellular trafficking and clear-up of Aβ, or the components or pathways that induce Aβ build-up or production [24-27]. The protective effect of the T carriers was seen also for SP coverage; however, it was only significant for moderate SP.

*CR1 *CC genotype carriers were associated with an increased risk of having sparse compared to no SP; however, the trend was not consistent for increasing coverage of SP (data not shown), which was also true for the analysis grouping the rare homozygote with the heterozygotes. This suggests the effect of *CR1 *is complex and not as straight-forward as increasing SP risk and requires further investigation.

The lack of robust and numerous associations with the GWAS SNPs and brain lesions, alongside the strong *APOE*ε4 results, questions the involvement of SP in AD pathology. It may be a coincidence that SP are found in AD brains with evidence suggesting that they may be a part of normal aging [16,17]. In light of this, SP treatments have so far failed to improve patients' cognitive abilities [35] and current theories are moving away from SP and suggest soluble oligomeric Aβ may be the culprit in AD [36-39]. The scarcity of associations may be due to the small number of cases with SP within the TASTY series (31.1%), resulting in low power; however, we have a 600 case-strong cohort, which revealed strong associations between *APOE *with SP. It may also be due to the low strength of these prior associations in the original studies, which should be investigated in future cohorts of a similar nature.

Some may question the use of an autopsy series to investigate an age-dependent illness such as AD; however, the TASTY cohort provides a unique view into the early midlife prevalence of well-defined neuropathological lesions, showing their common prevalence.

## Conclusions

We have an interesting window into the development of neuropathological lesions and their associations with AD-risk genes in the general population, and as far as we know, this is the first study of its kind. SP were found to associate with age, gender, and *APOE*ε4, but not consistently with *CLU*, *CR1 *or *PICALM*, suggesting that these SNPs most likely do not affect the development of the studied neuropathological lesions. Further studies should replicate these findings in a larger autopsy series of the same kind, both with and without AD cases, to define the occurrence of these neuropathological lesions within the context of normal aging.

Our results suggest that whilst these SNPs are associated with probable AD cases (in the GWAS), they do not strongly relate to SP prevalence, or at all to NFT, in an autopsy series most representative of the general population, possibly indicating their complex involvement in the disease.

## Abbreviations

AD: Alzheimer's disease; APOE: apolipoprotein E; CI: confidence interval; CLU: clusterin; CR1: complement component (3b/4b) receptor 1; GWAS: genome wide association studies; NFT: neurofibrillary tangles; OR: odds ratio; PICALM: phosphatidylinositol binding clathrin assembly protein; SNPs: single nucleotide polymorphisms; SP: senile plaques; TASTY: Tampere autopsy study.

## Competing interests

The authors declare that they have no competing interests.

## Authors' contributions

All authors contributed to this manuscript. EHK performed experiments and analyses and wrote the manuscript. HH, TL and SH measured the neuropathological lesions. SG and PJK collected the autopsy series. SG, HH and PJK provided comments and discussions on the progress of the manuscript.
